# (*E*)-[2-(1,3-Dithio­lan-2-yl­idene)hydrazinyl­idene](3-fluoro­phen­yl)methyl 3-fluoro­benzoate

**DOI:** 10.1107/S1600536813007319

**Published:** 2013-03-23

**Authors:** Ling Yin

**Affiliations:** a Department of Chemistry and Chemical Engineering, Jining University, Qufu 273155, People’s Republic of China

## Abstract

In the title compound, C_17_H_12_F_2_N_2_O_2_S_2_, the conformation of the dithia­cyclo­pentane ring is a half-chair, with a total puckering amplitude *Q*_T_ = 0.460 (1) Å. π–π inter­actions [centroid–centroid distance = 3.585 (9) Å between the fluoro­phenyl rings of neighbouring mol­ecules] and C—H⋯N and C—H⋯O inter­actions help to stabilize the crystal structure and form ladders along the *c* axis.

## Related literature

For the use of dithio­lan heterocyclic compounds as broad-spectrum fungicides, see: Tanaka *et al.* (1976 ▶); Wang *et al.* (1994 ▶).
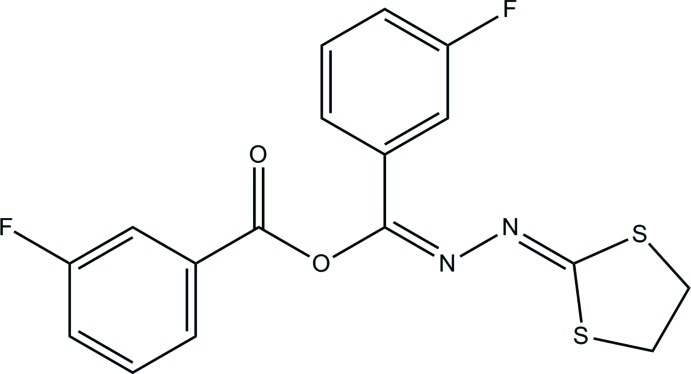


## Experimental

### 

#### Crystal data

C_17_H_12_F_2_N_2_O_2_S_2_
*M* *_r_* = 378.41
Triclinic, 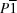

*a* = 9.124 (4) Å
*b* = 9.757 (4) Å
*c* = 10.738 (4) Å
α = 104.592 (3)°
β = 110.326 (5)°
γ = 101.194 (4)°
*V* = 824.6 (6) Å^3^
*Z* = 2
Mo *K*α radiation
μ = 0.36 mm^−1^
*T* = 113 K
0.20 × 0.20 × 0.20 mm

#### Data collection

Rigaku Saturn724 CCD diffractometer
Absorption correction: multi-scan (*CrystalClear*; Rigaku/MSC, 2009) ▶ *T*_min_ = 0.932, *T*_max_ = 0.932
7057 measured reflections
2896 independent reflections
2098 reflections with *I* > 2σ(*I*)
*R*_int_ = 0.036

#### Refinement

*R*[*F* ^2^ > 2σ(*F* ^2^)] = 0.031
*wR*(*F* ^2^) = 0.076
*S* = 0.96
2896 reflections
226 parameters
H-atom parameters constrained
Δρ_max_ = 0.39 e Å^−3^
Δρ_min_ = −0.20 e Å^−3^

### 

Data collection: *CrystalClear-SM Expert* (Rigaku/MSC, 2009) ▶; cell refinement: *CrystalClear-SM Expert*; data reduction: *CrystalClear-SM Expert*; program(s) used to solve structure: *SHELXS97* (Sheldrick, 2008 ▶); program(s) used to refine structure: *SHELXL97* (Sheldrick, 2008 ▶); molecular graphics: *SHELXTL* (Sheldrick, 2008 ▶); software used to prepare material for publication: *SHELXTL*.

## Supplementary Material

Crystal structure: contains datablock(s) I, global. DOI: 10.1107/S1600536813007319/hg5300sup1.cif

Structure factors: contains datablock(s) I. DOI: 10.1107/S1600536813007319/hg5300Isup2.hkl

Supplementary material file. DOI: 10.1107/S1600536813007319/hg5300Isup3.cml

Additional supplementary materials:  crystallographic information; 3D view; checkCIF report

## Figures and Tables

**Table 1:** Hydrogen-bond geometry (Å, °)

*D*—H⋯*A*	*D*—H	H⋯*A*	*D*⋯*A*	*D*—H⋯*A*
C9—H9*B*⋯O2^i^	0.97	2.49	3.280 (2)	138
C10—H10*B*⋯O2^ii^	0.97	2.46	3.248 (2)	138
C13—H13*A*⋯N1^iii^	0.93	2.50	3.424 (3)	176

Symmetry codes: (i) 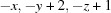; (ii) 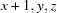; (iii) 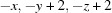.

**Fig. 1.**
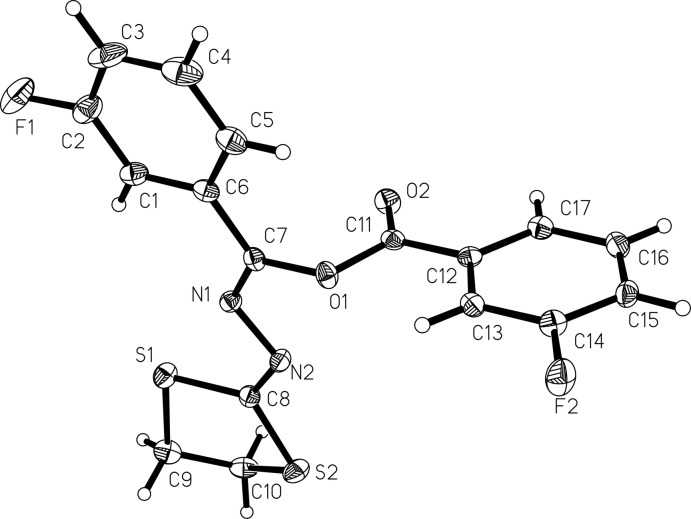
View of the title compound showing the atomic numbering and 40% probability displacement ellipsoids.

## supplementary crystallographic information

### Comment

Many dithiolan heterocyclic compounds have been widely used as potent and broad-spectrum fungicides (Tanaka *et al.*, 1976; Wang *et al.*, 1994). In order to search for new heterocylic compounds with higher biological activities, we synthesized the (*E*)-((1,3-dithiolan-2-yl)diazenyl)(3-fluorophenyl)methyl 3-fluorobenzoate and describe its structure here.

In the title compound, C_17_H_12_F_2_N_2_O_2_S_2_, the conformation of the dithiacyclopentane ring (C8—C10/S1—S2) is halfchair, with a total puckering amplitude Q_T_ = 0.460 (1) Å. π-π interactions (centroid-to-centroid distances 3.585 (9) Å between the fluorophenyl rings (C12—C17) of neighbouring molecules) and intermolecular C—H···N and C—H···O interactions help to stabilize the crystal structure (Table 1).

### Experimental

1.34 g (10 mmol) of (1,3-dithiolan-2-ylidene)hydrazine and 20 mmol triethylamine was dissolved in 15 ml of dichloromethane and stirred at room temperature, 3.17 g (20 mmol) 3-fluorobenzoyl chloride was added dropwise to the mixture. The reaction mixture was stirred vigorously at 273 K for 3 h. The reaction mixture was poured into 200 ml of water and extracted with three 50-ml portions of dichloromethane. The combined extracts were washed with saturated brine, dried over anhydrous sodium sulfate and evaporated on a rotary evaporator to afford the crude product, which was purified by column chromatography to yield the pure product as colorless crystals. Single crystals suitable for X-ray diffraction were obtained through slow evaporation of a solution of the pure title compound in ethanol.

### Refinement

All H atoms bonded on carbon were found on difference maps, with C–H = 0.93 or 0.97 Å, and included in the final cycles of refinement using a riding model, with *U*_iso_(H) = 1.2*U*_eq_(C).

### Crystal data

**Table:**

C_17_H_12_F_2_N_2_O_2_S_2_	*Z* = 2
*M**_r_* = 378.41	*F*(000) = 388
Triclinic, *P*1	*D*_x_ = 1.524 Mg m^−^^3^
Hall symbol: -P 1	Mo *K*α radiation, λ = 0.71073 Å
*a* = 9.124 (4) Å	Cell parameters from 2617 reflections
*b* = 9.757 (4) Å	θ = 2.2–27.9°
*c* = 10.738 (4) Å	µ = 0.36 mm^−^^1^
α = 104.592 (3)°	*T* = 113 K
β = 110.326 (5)°	Block, colorless
γ = 101.194 (4)°	0.20 × 0.20 × 0.20 mm
*V* = 824.6 (6) Å^3^	

### Data collection

**Table:**

Rigaku Saturn724 CCD diffractometer	2896 independent reflections
Radiation source: rotating anode	2098 reflections with *I* > 2σ(*I*)
Multilayer monochromator	*R*_int_ = 0.036
Detector resolution: 14.22 pixels mm^-1^	θ_max_ = 25.0°, θ_min_ = 2.2°
ω and φ scans	*h* = −10→10
Absorption correction: multi-scan (*CrystalClear*; Rigaku/MSC, 2009)	*k* = −11→11
*T*_min_ = 0.932, *T*_max_ = 0.932	*l* = −12→12
7057 measured reflections	

### Refinement

**Table:**

Refinement on *F*^2^	Primary atom site location: structure-invariant direct methods
Least-squares matrix: full	Secondary atom site location: difference Fourier map
*R*[*F*^2^ > 2σ(*F*^2^)] = 0.031	Hydrogen site location: inferred from neighbouring sites
*wR*(*F*^2^) = 0.076	H-atom parameters constrained
*S* = 0.96	*w* = 1/[σ^2^(*F*_o_^2^) + (0.0322*P*)^2^] where *P* = (*F*_o_^2^ + 2*F*_c_^2^)/3
2896 reflections	(Δ/σ)_max_ = 0.001
226 parameters	Δρ_max_ = 0.39 e Å^−^^3^
0 restraints	Δρ_min_ = −0.20 e Å^−^^3^

### Special details

**Table:**

Geometry. All e.s.d.'s (except the e.s.d. in the dihedral angle between two l.s. planes) are estimated using the full covariance matrix. The cell e.s.d.'s are taken into account individually in the estimation of e.s.d.'s in distances, angles and torsion angles; correlations between e.s.d.'s in cell parameters are only used when they are defined by crystal symmetry. An approximate (isotropic) treatment of cell e.s.d.'s is used for estimating e.s.d.'s involving l.s. planes.
Refinement. Refinement of *F*^2^ against ALL reflections. The weighted *R*-factor *wR* and goodness of fit *S* are based on *F*^2^, conventional *R*-factors *R* are based on *F*, with *F* set to zero for negative *F*^2^. The threshold expression of *F*^2^ > σ(*F*^2^) is used only for calculating *R*-factors(gt) *etc*. and is not relevant to the choice of reflections for refinement. *R*-factors based on *F*^2^ are statistically about twice as large as those based on *F*, and *R*- factors based on ALL data will be even larger.

### Fractional atomic coordinates and isotropic or equivalent isotropic displacement parameters (Å^2^)

**Table:**

	*x*	*y*	*z*	*U*_iso_*/*U*_eq_	
S1	0.10124 (6)	0.83442 (5)	0.59445 (5)	0.02517 (15)	
O1	−0.12238 (14)	1.08666 (13)	0.92674 (12)	0.0209 (3)	
N1	−0.06721 (17)	0.92577 (15)	0.75780 (14)	0.0183 (4)	
F1	−0.49133 (14)	0.43408 (13)	0.61224 (12)	0.0447 (4)	
C1	−0.3309 (2)	0.6858 (2)	0.71390 (18)	0.0234 (5)	
H1B	−0.2714	0.6668	0.6602	0.028*	
S2	0.31536 (6)	1.14801 (5)	0.73274 (5)	0.02773 (15)	
F2	0.06577 (13)	1.50851 (11)	1.36811 (11)	0.0320 (3)	
O2	−0.27848 (15)	1.15266 (14)	0.75200 (12)	0.0241 (3)	
N2	0.05995 (17)	1.05067 (16)	0.78311 (15)	0.0205 (4)	
C2	−0.4583 (2)	0.5746 (2)	0.7016 (2)	0.0300 (5)	
C3	−0.5519 (2)	0.5965 (2)	0.7762 (2)	0.0378 (6)	
H3A	−0.6389	0.5187	0.7637	0.045*	
C4	−0.5130 (2)	0.7376 (3)	0.8706 (2)	0.0375 (6)	
H4A	−0.5742	0.7552	0.9231	0.045*	
C5	−0.3846 (2)	0.8530 (2)	0.88806 (19)	0.0277 (5)	
H5A	−0.3591	0.9474	0.9524	0.033*	
C6	−0.2929 (2)	0.8280 (2)	0.80908 (18)	0.0194 (4)	
C7	−0.1555 (2)	0.94860 (19)	0.82551 (17)	0.0177 (4)	
C8	0.1433 (2)	1.01277 (19)	0.71174 (18)	0.0196 (4)	
C9	0.2838 (2)	0.8807 (2)	0.56131 (19)	0.0281 (5)	
H9A	0.3748	0.8649	0.6302	0.034*	
H9B	0.2634	0.8176	0.4675	0.034*	
C10	0.3251 (2)	1.0425 (2)	0.57270 (19)	0.0273 (5)	
H10A	0.2471	1.0544	0.4909	0.033*	
H10B	0.4345	1.0771	0.5771	0.033*	
C11	−0.1953 (2)	1.18274 (19)	0.87559 (19)	0.0183 (4)	
C12	−0.1586 (2)	1.32341 (19)	0.99052 (18)	0.0176 (4)	
C13	−0.0640 (2)	1.3463 (2)	1.13176 (18)	0.0193 (4)	
H13A	−0.0256	1.2719	1.1584	0.023*	
C14	−0.0294 (2)	1.4828 (2)	1.23034 (18)	0.0214 (4)	
C15	−0.0852 (2)	1.5955 (2)	1.19552 (19)	0.0219 (4)	
H15A	−0.0584	1.6869	1.2650	0.026*	
C16	−0.1816 (2)	1.5698 (2)	1.05533 (19)	0.0235 (5)	
H16A	−0.2215	1.6441	1.0299	0.028*	
C17	−0.2192 (2)	1.4342 (2)	0.95261 (19)	0.0211 (4)	
H17A	−0.2847	1.4171	0.8584	0.025*	

### Atomic displacement parameters (Å^2^)

**Table:**

	*U*^11^	*U*^22^	*U*^33^	*U*^12^	*U*^13^	*U*^23^
S1	0.0288 (3)	0.0228 (3)	0.0234 (3)	0.0054 (2)	0.0145 (2)	0.0038 (2)
O1	0.0265 (7)	0.0204 (7)	0.0161 (7)	0.0112 (6)	0.0082 (6)	0.0048 (6)
N1	0.0181 (8)	0.0184 (8)	0.0171 (8)	0.0044 (7)	0.0065 (7)	0.0062 (7)
F1	0.0460 (8)	0.0304 (7)	0.0372 (7)	−0.0090 (6)	0.0073 (6)	0.0089 (6)
C1	0.0196 (10)	0.0296 (11)	0.0191 (10)	0.0050 (9)	0.0059 (9)	0.0106 (9)
S2	0.0240 (3)	0.0262 (3)	0.0290 (3)	0.0009 (2)	0.0132 (2)	0.0055 (2)
F2	0.0423 (7)	0.0278 (6)	0.0184 (6)	0.0112 (5)	0.0067 (5)	0.0041 (5)
O2	0.0255 (7)	0.0278 (7)	0.0168 (7)	0.0102 (6)	0.0054 (6)	0.0076 (6)
N2	0.0213 (9)	0.0163 (8)	0.0221 (8)	0.0036 (7)	0.0093 (7)	0.0052 (7)
C2	0.0285 (12)	0.0274 (11)	0.0230 (11)	0.0003 (9)	0.0028 (10)	0.0088 (10)
C3	0.0219 (12)	0.0488 (15)	0.0404 (13)	−0.0011 (11)	0.0090 (11)	0.0265 (12)
C4	0.0287 (12)	0.0591 (16)	0.0381 (13)	0.0155 (11)	0.0211 (11)	0.0265 (12)
C5	0.0259 (11)	0.0374 (12)	0.0253 (11)	0.0146 (10)	0.0111 (10)	0.0156 (10)
C6	0.0178 (10)	0.0271 (11)	0.0146 (10)	0.0087 (8)	0.0051 (8)	0.0101 (9)
C7	0.0204 (10)	0.0196 (10)	0.0119 (9)	0.0090 (8)	0.0040 (8)	0.0056 (8)
C8	0.0211 (10)	0.0195 (10)	0.0166 (10)	0.0067 (8)	0.0050 (9)	0.0074 (8)
C9	0.0277 (12)	0.0366 (12)	0.0230 (11)	0.0118 (10)	0.0136 (10)	0.0092 (10)
C10	0.0207 (10)	0.0397 (12)	0.0225 (11)	0.0069 (9)	0.0100 (9)	0.0130 (10)
C11	0.0157 (10)	0.0218 (10)	0.0224 (11)	0.0063 (8)	0.0110 (9)	0.0111 (9)
C12	0.0149 (10)	0.0203 (10)	0.0200 (10)	0.0037 (8)	0.0102 (8)	0.0080 (8)
C13	0.0195 (10)	0.0205 (10)	0.0227 (10)	0.0079 (8)	0.0110 (9)	0.0106 (9)
C14	0.0228 (11)	0.0244 (11)	0.0169 (10)	0.0065 (9)	0.0090 (9)	0.0065 (9)
C15	0.0238 (11)	0.0172 (10)	0.0254 (11)	0.0050 (8)	0.0141 (9)	0.0041 (9)
C16	0.0245 (11)	0.0217 (10)	0.0301 (11)	0.0103 (9)	0.0150 (10)	0.0110 (9)
C17	0.0204 (10)	0.0242 (10)	0.0222 (10)	0.0073 (8)	0.0102 (9)	0.0114 (9)

### Geometric parameters (Å, º)

**Table:**

S1—C8	1.7499 (19)	C5—C6	1.395 (2)
S1—C9	1.8178 (18)	C5—H5A	0.9300
O1—C11	1.368 (2)	C6—C7	1.469 (2)
O1—C7	1.401 (2)	C9—C10	1.513 (3)
N1—C7	1.274 (2)	C9—H9A	0.9700
N1—N2	1.4036 (19)	C9—H9B	0.9700
F1—C2	1.366 (2)	C10—H10A	0.9700
C1—C2	1.370 (2)	C10—H10B	0.9700
C1—C6	1.394 (2)	C11—C12	1.483 (2)
C1—H1B	0.9300	C12—C17	1.392 (3)
S2—C8	1.7480 (18)	C12—C13	1.393 (2)
S2—C10	1.8088 (19)	C13—C14	1.374 (2)
F2—C14	1.357 (2)	C13—H13A	0.9300
O2—C11	1.200 (2)	C14—C15	1.378 (3)
N2—C8	1.294 (2)	C15—C16	1.381 (2)
C2—C3	1.370 (3)	C15—H15A	0.9300
C3—C4	1.381 (3)	C16—C17	1.382 (2)
C3—H3A	0.9300	C16—H16A	0.9300
C4—C5	1.380 (3)	C17—H17A	0.9300
C4—H4A	0.9300		
			
C8—S1—C9	95.19 (8)	S1—C9—H9A	110.1
C11—O1—C7	115.41 (14)	C10—C9—H9B	110.1
C7—N1—N2	115.89 (14)	S1—C9—H9B	110.1
C2—C1—C6	118.25 (17)	H9A—C9—H9B	108.4
C2—C1—H1B	120.9	C9—C10—S2	107.36 (12)
C6—C1—H1B	120.9	C9—C10—H10A	110.2
C8—S2—C10	94.44 (8)	S2—C10—H10A	110.2
C8—N2—N1	110.12 (14)	C9—C10—H10B	110.2
F1—C2—C1	118.15 (17)	S2—C10—H10B	110.2
F1—C2—C3	118.45 (17)	H10A—C10—H10B	108.5
C1—C2—C3	123.39 (19)	O2—C11—O1	122.43 (17)
C2—C3—C4	117.95 (18)	O2—C11—C12	125.87 (18)
C2—C3—H3A	121.0	O1—C11—C12	111.70 (15)
C4—C3—H3A	121.0	C17—C12—C13	120.61 (17)
C5—C4—C3	120.84 (18)	C17—C12—C11	117.79 (17)
C5—C4—H4A	119.6	C13—C12—C11	121.59 (18)
C3—C4—H4A	119.6	C14—C13—C12	117.59 (18)
C4—C5—C6	119.96 (18)	C14—C13—H13A	121.2
C4—C5—H5A	120.0	C12—C13—H13A	121.2
C6—C5—H5A	120.0	F2—C14—C13	118.49 (18)
C1—C6—C5	119.59 (17)	F2—C14—C15	118.47 (16)
C1—C6—C7	119.26 (15)	C13—C14—C15	123.03 (18)
C5—C6—C7	121.15 (16)	C14—C15—C16	118.59 (17)
N1—C7—O1	122.72 (15)	C14—C15—H15A	120.7
N1—C7—C6	121.68 (16)	C16—C15—H15A	120.7
O1—C7—C6	115.47 (14)	C15—C16—C17	120.32 (19)
N2—C8—S2	118.67 (14)	C15—C16—H16A	119.8
N2—C8—S1	125.76 (13)	C17—C16—H16A	119.8
S2—C8—S1	115.57 (10)	C16—C17—C12	119.84 (18)
C10—C9—S1	108.17 (13)	C16—C17—H17A	120.1
C10—C9—H9A	110.1	C12—C17—H17A	120.1
			
C7—N1—N2—C8	178.22 (16)	C10—S2—C8—S1	17.80 (12)
C6—C1—C2—F1	−177.56 (17)	C9—S1—C8—N2	−174.67 (17)
C6—C1—C2—C3	1.1 (3)	C9—S1—C8—S2	4.65 (12)
F1—C2—C3—C4	177.36 (18)	C8—S1—C9—C10	−31.34 (14)
C1—C2—C3—C4	−1.3 (3)	S1—C9—C10—S2	46.97 (15)
C2—C3—C4—C5	0.5 (3)	C8—S2—C10—C9	−38.84 (15)
C3—C4—C5—C6	0.4 (3)	C7—O1—C11—O2	−1.9 (2)
C2—C1—C6—C5	−0.1 (3)	C7—O1—C11—C12	177.88 (12)
C2—C1—C6—C7	179.21 (16)	O2—C11—C12—C17	−3.1 (3)
C4—C5—C6—C1	−0.6 (3)	O1—C11—C12—C17	177.18 (14)
C4—C5—C6—C7	−179.95 (17)	O2—C11—C12—C13	178.22 (16)
N2—N1—C7—O1	−4.3 (2)	O1—C11—C12—C13	−1.5 (2)
N2—N1—C7—C6	179.92 (15)	C17—C12—C13—C14	−1.8 (2)
C11—O1—C7—N1	89.6 (2)	C11—C12—C13—C14	176.82 (14)
C11—O1—C7—C6	−94.45 (18)	C12—C13—C14—F2	−178.49 (14)
C1—C6—C7—N1	−1.9 (3)	C12—C13—C14—C15	0.6 (3)
C5—C6—C7—N1	177.38 (17)	F2—C14—C15—C16	179.79 (15)
C1—C6—C7—O1	−177.96 (16)	C13—C14—C15—C16	0.7 (3)
C5—C6—C7—O1	1.4 (2)	C14—C15—C16—C17	−0.8 (3)
N1—N2—C8—S2	−176.83 (12)	C15—C16—C17—C12	−0.4 (3)
N1—N2—C8—S1	2.5 (2)	C13—C12—C17—C16	1.8 (2)
C10—S2—C8—N2	−162.83 (16)	C11—C12—C17—C16	−176.95 (15)

### Hydrogen-bond geometry (Å, º)

**Table:**

*D*—H···*A*	*D*—H	H···*A*	*D*···*A*	*D*—H···*A*
C9—H9*B*···O2^i^	0.97	2.49	3.280 (2)	138
C10—H10*B*···O2^ii^	0.97	2.46	3.248 (2)	138
C13—H13*A*···N1^iii^	0.93	2.50	3.424 (3)	176

Symmetry codes: (i) −*x*, −*y*+2, −*z*+1; (ii) *x*+1, *y*, *z*; (iii) −*x*, −*y*+2, −*z*+2.
